# High-Throughput Predictions of the Stabilities of Multi-Type Long-Period Stacking Ordered Structures in High-Performance Mg Alloys

**DOI:** 10.3390/nano12183240

**Published:** 2022-09-18

**Authors:** Touwen Fan, Zhipeng Wang, Yuanyuan Tian, Yu Liu, Pingying Tang

**Affiliations:** 1 College of Science, Hunan Institute of Technology, Hengyang 421002, China; 2 School of Mechanical and Electrical Engineering, Central South University of Forestry and Technology, Changsha 410004, China; 3 State Key Laboratory of Advanced Design and Manufacturing for Vehicle Body, Hunan University, Changsha 410082, China; 4 School of Mechanical and Aerospace Engineering, Nanyang Technological University, 50 Nanyang Avenue, Singapore 639798, Singapore; 5 College of Materials Science and Engineering, Hunan University, Changsha 410082, China; 6 Key Laboratory of New Electric Functional Materials of Guangxi Colleges and Universities, Nanning Normal University, Nanning 530023, China

**Keywords:** Mg alloys, long-period stacking-ordered structures, stability, first-principles calculations, solute atoms

## Abstract

The effects of 44 types of elements on the stabilities of I1-constitute multi-type long-period stacking-ordered (LPSO) structures in Mg alloys, such as 4H, 6H, 8H, 9R, 12H, 15R, and 16H phases, are systematically investigated by first-principle high-performance calculations. The intrinsic stacking-fault energies (ISFEs) and their increments are calculated along with the formation enthalpies of solute atoms, and interaction energies between solute atoms and LPSO structures. The results suggest that the 15R phase is the easiest to form and stabilize among these LPSO structures, and 44 types of solute atoms have different segregation characteristics in these LPSO structures. A high temperature inhibits structural stabilizations of the LPSO phases, and these alloying elements, such as elements (Sb, Te, and Cs) for 4H; elements (S, Fe, Sb, and Te) for 6H, 8H, 9R, 15R, and 16H; and elements (S, Sb, and Te) for 12H, can effectively promote the stability of LPSO structures at high temperatures. S and Fe atoms are the most likely to promote the stabilities of the 16H structure with regard to other LPSO phases, but the Fe atom tends to inhibit the stabilities of 4H and 12H structures. This work can offer valuable references to further study and develop high-performance Mg alloys with multi-type LPSO structures.

## 1. Introduction

Magnesium (Mg) is regarded as one of the lightest metals due to its low density [1]. Mg alloys exhibit excellent comprehensive properties, including high strength, good machinability and thermal conductivity, strong electromagnetic shielding capability, and favorable biocompatibility and biodegradability. They have been widely used in automobile, architecture, transportation, biomedical, electronics, and other fields [2,3,4,5]. To date, plenty of technologies, including heat treatment [6], alloying [7], selective laser melting [8], severe plastic deformation [9,10], magnetron sputtering [11], and sputter deposition [12], have been used in developing Mg alloys with high performance. In a 3.5 wt.% NaCl solution saturated with Mg(OH)_2_, Cao et al. [6] studied the corrosion behavior of rolled Mg5Gd alloys under heat treatment, and the results revealed that the corrosion rate of the Mg5Gd dramatically decreased under a solution treatment, and the corrosion rate could be further reduced in the following aging process. Chen et al. [7] investigated the microstructure and tensile properties as a function of Sn content in as-cast and as-extruded Mg–8Li–3Al–(1,2,3)Sn alloys and found that increasing Sn content contributed to improving the strength of as-cast Mg–8Li–3Al–(1,2,3)Sn alloys by grain refinement, which was helpful to prepare Mg alloys of high strength. With the aid of selective laser melting technology, Gao et al. [8] observed that the dual alloying effects of Mn and/or Sn induced remarkable refinement of both the grains and the intermetallic phases in the process of rapid solidification and led to a drop of nearly half in the corrosion rate for the AZ61–0.4Mn–0.8Sn alloy, as well as effective improvement of strength and hardness, and these findings promoted the applications of AZ61–Mn–Sn alloy in biodegradable bone implants. In addition, severe plastic deformation, magnetron sputtering, and sputter-deposited technologies are also broadly used for fabricating Mg alloys of exceptional properties, including high strength, high hardness, outstanding anti-corrosion, excellent anti-fatigue, anti-wear, etc. [9,10,11,12,13,14,15]. Alloying is also an effective way to design high-performance Mg alloys by adjusting their SFEs, which has been confirmed by other works [16,17,18,19].

In the process of studying Mg alloys with high performances, LPSO structures, such as the 4H, 6H, 8H, 9R, 10H, 12H, 14H, 15R, 16H, 18R, and 24R phases [20,21,22,23], are considered special phase structures in enhancing the mechanical properties of Mg alloys, and they have generally been observed in Mg alloys because of the low stacking fault energy (SFE) ($\gamma_{\mathrm{SFE}}=33.84\;\mathrm{mJ}/m^{2}$) [24,25,26,27]. With the help of atomic-resolution Z-contrast STEM, Abe et al. [20] found that adding a few Zn and Y elements in Mg promoted the formation of a 6H-type LPSO structure, which was ascribed to Zn and Y atoms adjacent to the faulting layers, and then effectively improved the nucleation and growth of the 6H-type LPSO structure. By transmission electron microscopy (TEM) and scanning transmission electron microscopy (STEM), Mi and Jin [26] found new polytypes of LPSO structures in Mg–Co–Y alloys, including 15R-, 12H-, and 21R-type LPSO structures. The Co/Y element was segregated to the stacking layers in these LPSO structures, promoting the stability of the LPSO structures, but the stabilization mechanism is still unknown. Huang et al. [28] studied the effects of Sn on the formation of the LPSO phase and mechanical properties of Mg–RE–Zn alloy, and the doping Sn atom induced a high density of the lamellar-shaped 14H-type LPSO phase, and the addition of Sn atom enhanced the strength of the alloy despite a certain decrease in the elongation. Recently, six new polytypes of LPSO structures including 29H, 51R, 60H, 72R, 102R, and 192R, have also been discovered in the Mg_92_Co_2_Y_6_ alloy [27], which further enriches the members of LPSO structures and provides the possibility for enhancing mechanical properties of Mg alloys. However, the stabilization mechanisms of these LPSO structures are still unclear, thus restricting the development of high-performance Mg alloys.

Based on the above problems, this work applies first-principle high-performance calculations to investigate the stabilization mechanisms of multi-type LPSO structures in Mg alloys, and the effects of 44 types of elements (Li, Be, B, Na, Al, Si, P, S, K, Ca, Sc, Ti, V, Cr, Mn, Fe, Co, Ni, Cu, Zn, Ga, Ge, Se, Sr, Y, Zr, Nb, Mo, Ag, Cd, Sn, Sb, Te, Cs, Ba, Hf, Ta, W, Pt, Au, Pb, Bi, Ru, and Re) from a periodic table of elements on the stabilities of the LPSO structures. We calculate the formation enthalpies of solute atoms and their interaction energies with the LPSO structures. The Fermi–Dirac distribution (FDD) model is used for describing solute distributions in the LPSO phases and to investigate the increments of ISFEs as a function of solute concentration and temperature. In this work, the general framework in the remaining sections is organized as follows: Section 2 depicts the theoretical models of the I1-constitute LPSO structures; Section 3 introduces the first-principles method in all calculations of this work; Section 4 analyzes the calculated results, which agree well with the available experimental data and other calculated results; and Section 5 summarizes the relevant conclusions of this work.

## 2. Theoretical Model

**Figure 1:**
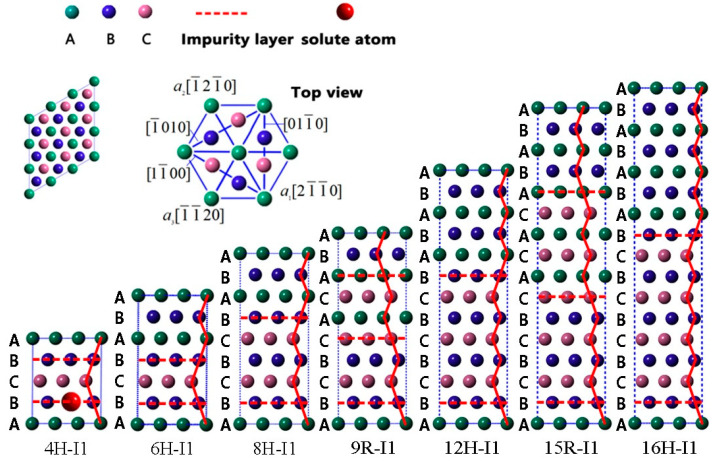
The I1-constitute LPSO structures for 4H, 6H, 8H, 9R, 12H, 15R, and 16H phases.

According to previous works [24,29,30], LPSO phases are multi-stacking fault structures classified into two groups of I1 and I2 plane faults, and each LPSO structure consists of the same type of stacking faults. Herein, we study the stabilities of LPSO structures with I1 plane fault, such as 4H, 6H, 8H, 9R, 12H, 15R, and 16H, and compare their relative stabilities by doping solute atoms. In Mg with hexagonal close-packed (HCP) structure…, ABABABABABAB… is the perfect stacking sequence of (0001) basal plane in the direction of [0001]. With regard to the 4H, 6H, 8H, 9R, 12H, 15R, and 16H structures, their atomic sequences are severally transformed as follows: …ABCBA…, …ABCBABA…, …ABCBCBABA…, …ABCBCACABA…, …ABCBCBCBABABA…, …ABCBCBCACACABABA…, …ABCBCBCBCBABABABA…, where A, B, and C indicate different (0001) basal planes. Figure 1 depicts severally the seven theoretical models of 4H, 6H, 8H, 9R, 12H, 15R, and 16H I1-constitute LPSO structures, and the doped solute atoms are located in various atomic layers of these LPSO structures.

The variables $\gamma_{m-\mathrm{Mg}}$ (m=4H,6H,8H,9R,12H,15R,16H) are defined as energy differences per area caused by the corresponding structural phase, and they can be formulized as follows:

(1) $$\;\gamma_{m-\mathrm{Mg}}=\left[E_{m-\mathrm{Mg}}-E_{m-perf-\mathrm{Mg}}\right]/A_{m-perf}$$

where $E_{m-\mathrm{Mg}}$ denotes the total energy of m phases and $E_{m-perf-\mathrm{Mg}}$ indicates one of the corresponding perfect supercells in Mg metal. $A_{m-perf}$ is the (0001) area of the perfect supercell. This work dopes 44 types of solute atoms (Li, Be, B, Na, Al, Si, P, S, K, Ca, Sc, Ti, V, Cr, Mn, Fe, Co, Ni, Cu, Zn, Ga, Ge, Se, Sr, Y, Zr, Nb, Mo, Ag, Cd, Sn, Sb, Te, Cs, Ba, Hf, Ta, W, Pt, Au, Pb, Bi, Ru, and Re) from the periodic table of elements in different atomic layers of m phases, and then, the temperature-dependent ISFEs $\gamma_{m}(T)$ can be calculated as follows [31,32]:

(2) $$\;\gamma_{m}(T)=\gamma_{m-\mathrm{Mg}}+\sum_{n}c_{m-n}(T)E_{int-m-n}/{A^{\prime }}_{m-perf}$$

where ${A^{\prime }}_{m-perf}$ indicates the area of the (0001) unit cell and $c_{m-n}(T)$ denotes solute concentration in the *n*-th atomic layer of *m* phases at temperature *T*. $E_{int-m-n}$ is the interaction energy between solute atoms of *n*-th layer and *m* phases, which can be calculated as follows [31]:

(3) $$\;E_{int-m-n}=\left[E_{s-m-n}-E_{s-perf-\mathrm{Mg}}\right]-\left[E_{m-\mathrm{Mg}}-E_{m-perf-\mathrm{Mg}}\right]$$

where $E_{s-m-n}$ is the total energy of *m* phases doped by a solute atom in the *n*-th layer and $E_{s-perf-\mathrm{Mg}}$ is the one of the corresponding perfect supercells doped by a solute atom, respectively.

The FDD model is introduced to investigate the effects of solute concentration and finite temperature on the ISFE and its increment of *m* phases. In light of the FDD model, the solute concentration $c_{m-n}(T)$ in the *n*-th layer can be expressed as follows [31,33,34,35]:

(4) $$c_{m-n}(T)=\frac{1}{1+\exp\left(\frac{E_{int-m-n}}{kT}-\ln\frac{c_{0}}{1-c_{0}}\right)}$$

Herein, *k* denotes the Boltzmann constant and $c_{0}$ is the solute concentration. Due to common effects of solute concentration and temperature, we deduce the increments $\Delta \gamma_{m}(T)$ of ISFEs as follows:

(5) $$\Delta \gamma_{m}(T)=\sum_{n}c_{m-n}(T)E_{int-m-n}/{A^{\prime }}_{m-perf}$$

## 3. Methodology

All DFT calculations in this work were executed based on the Vienna ab initio simulation package (VASP) [36,37]. The ion–electron interactions were handled by the projector–augmented wave (PAW) method [38]. The generalized gradient approximation (GGA) of Perdew–Burke–Eruzerhof (PBE) [39] is defined as the exchange-correlation functional. To study the stability of *m* phases, we built 3 × 3 × 4 for 4H, 3 × 3 × 6 for 6H, 3 × 3 × 8 for 8H, 3 × 3 × 9 for 9R, 3 × 3 × 12 for 12H, 3 × 3 × 15 for 15R, and 3 × 3 × 16 for 16H supercells (see Figure 1), aiming to obtain the interaction energies of solute atoms with LPSO structures. Before the DFT calculations, we conducted strict convergence tests to ensure sufficient calculation accuracy, and the obtained optimization parameter for cutoff energy of plane wave basis was 350 eV. k-mesh Gamma-centered Monkhorst–Pack grids [40] in Brillouin zone sampling were optimized as 5 × 5 × 5 for 4H, 5 × 5 × 5 for 6H, 5 × 5 × 2 for 8H, 5 × 5 × 1 for 9R, 5 × 5 × 1 for 12H, 5 × 5 × 1 for 15R, and 5 × 5 × 1 for 16H. The Hellmann–Feyman force acting on each atom was less than 0.01 eV/Å, and the total energy of self-consistent calculation was precisely converged as 10^−6^ eV/atom.

## 4. Results and Discussion

**Figure 2:**
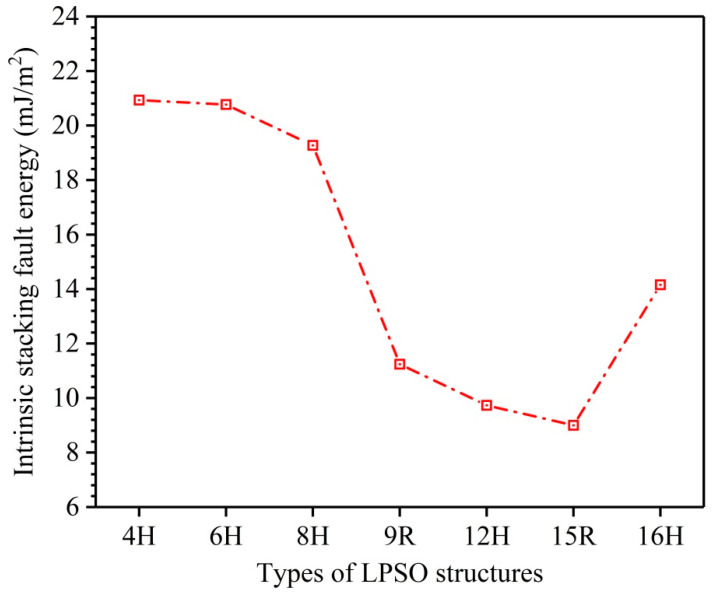
Comparisons of calculated ISFEs for 4H, 6H, 8H, 9R, 12H, 15R, and 16H phases.

Due to low SFE, many kinds of LPSO structures, such as the 4H, 6H, 8H, 9R, 10H, 12H, 14H, 15R, 16H, 18R, 24R, 29H, 51R, 60H, 72R, 102R, and 192R phases, etc. [20,21,22,23,27], are easy to form and exist stably in Mg metal and its alloys. Herein, I1-constitute LPSO structures including 4H, 6H, 8H, 9R, 12H, 15R, and 16H are investigated, and the calculated results of their ISFEs are shown in Figure 2. The values of these ISFEs are calculated as follows: 20.93 mJ/m^2^ for 4H, 20.77 mJ/m^2^ for 6H, 19.27 mJ/m^2^ for 8H, 11.24 mJ/m^2^ for 9R, 9.73 mJ/m^2^ for 12H, 9.00 mJ/m^2^ for 15R, and 14.16 mJ/m^2^ for 16H. According to the calculated results of the ISFEs, the order of their numerical sizes is as follows: 4H > 6H > 8H > 16H > 9R > 12H > 15R, indicating that these LPSO structures for 9R, 12H, 15R, and 16H are essentially easy to form and exist stably in Mg metal due to low ISFEs, and the 15R phase is the easiest to form and stabilize among these LPSO structures because of the lowest ISFE, but the others for 4H, 6H, and 8H are comparatively difficult to form and exist stably owing to their relatively high ISFEs, which agrees well with calculation results of formation energy [41] and reveals the reasons for hardly discovering I1-constitute 4H, 6H, and 8H phases in Mg.

**Figure 3:**
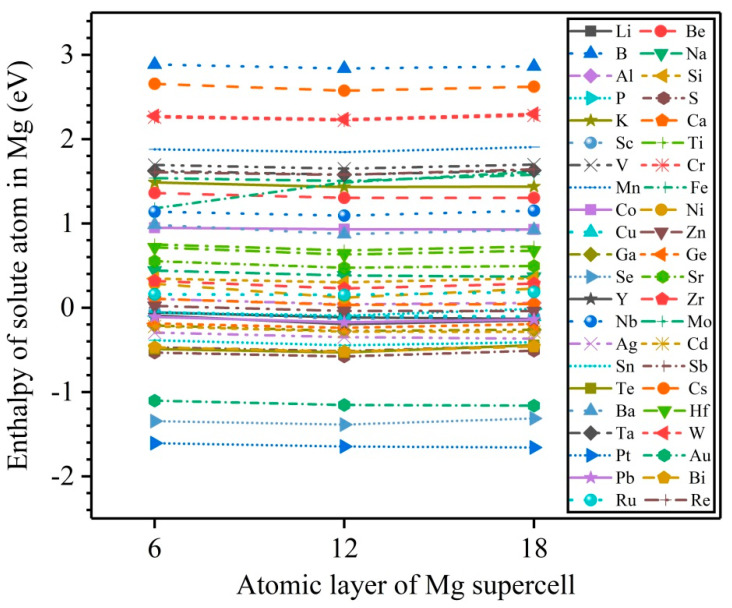
The calculated results of formation enthalpies of solute atoms in a 3 × 3 × L (L = 6, 12, 18) magnesium perfect supercell with one magnesium substituted by a solute.

Formation enthalpy is an important parameter to evaluate the solubility of solute atoms in a solid solution. The 3 × 3 × L (L = 6, 12, 18) magnesium perfect supercells are constructed to calculate the formation enthalpies of 44 types of solute atoms, corresponding to the bulk solute concentrations of 1.85, 0.93, and 0.62 at.%. Figure 3 exhibits the calculated results. Obviously, the formation enthalpies of 44 types of solute atoms remain almost constant with an increasing solute concentration, indicating that the solute concentration slightly affects the formation enthalpy of solute atoms in Mg bulk. In addition, Figure 3 also shows that 25 types of solute atoms (Be, B, Na, Al, Si, K, Ca, Ti, V, Cr, Mn, Fe, Co, Ni, Sr, Zr, Nb, Mo, Cs, Ba, Hf, Ta, W, Ru, and Re) have positive formation enthalpies, implying that these solute atoms are dissolved in Mg bulk with relative difficulty, while the remaining 19 types of solute atoms (Li, P, S, Sc, Cu, Zn, Ga, Ge, Se, Y, Ag, Cd, Sn, Sb, Te, Pt, Au, Pb, and Bi) have negative formation enthalpies, suggesting that they are easily dissolved in Mg bulk, and form Mg alloys.

**Figure 4:**
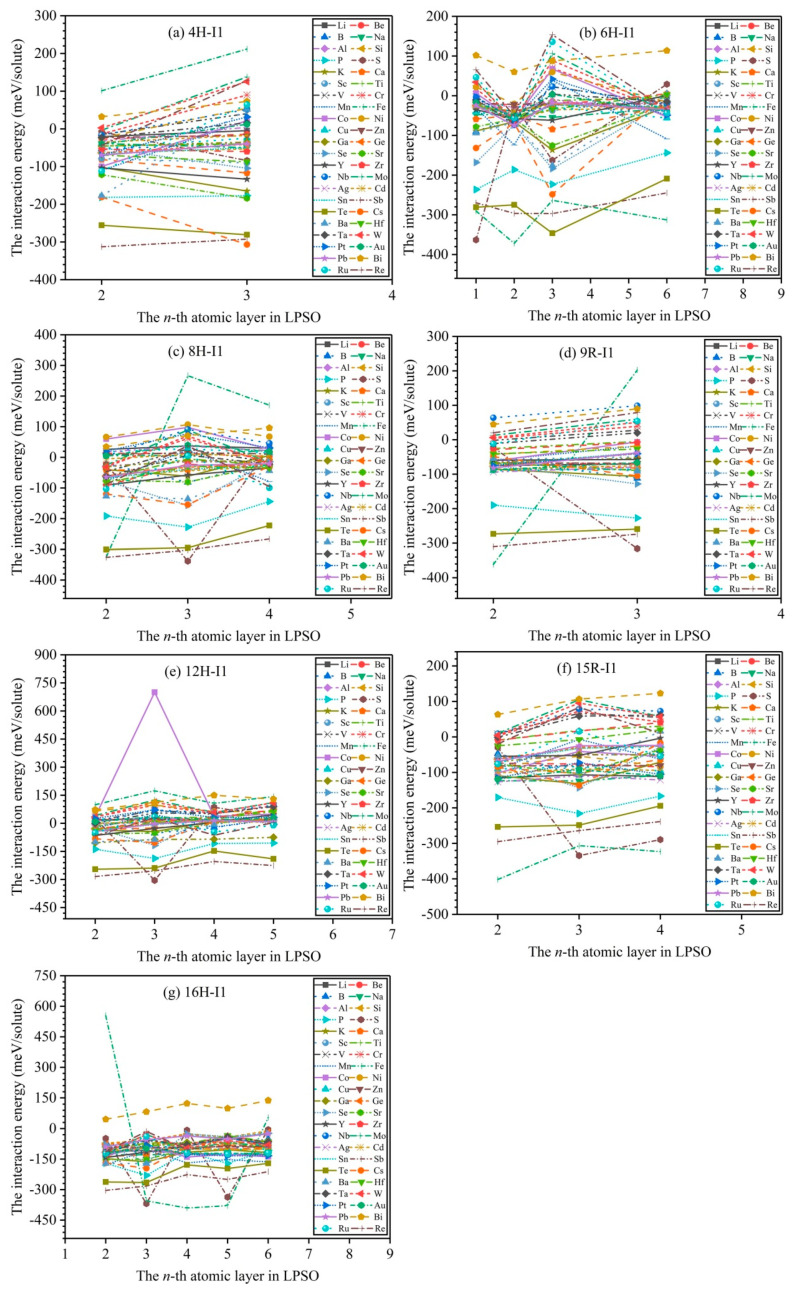
The calculated results of interaction energies between solute atoms in *n*-th layer and *m* phases: (**a**–**g**) denote 4H, 6H, 8H, 9R, 12H, 15R, and 16H phases, respectively.

Figure 4 exhibits the calculated results of interaction energies between solute atoms in the *n*-th layer and *m* phases. According to the structural symmetry of *m* phases (see Figure 1), we divide atomic layers with different chemical environmental characteristics as follows: two different atomic layers (second and third layers) for 4H, four different atomic layers (first, second, third, and sixth layers) for 6H, three different atomic layers (second, third, and fourth layers) for 8H, two different atomic layers (second and third layers) for 9R, four different atomic layers (second, third, fourth, and fifth layers) for 12H, three different atomic layers (second, third, and fourth layers) for 15R, and five different atomic layers (second, third, fourth, fifth, and sixth layers) for 16H. Herein, we dope solute atoms in the aforementioned atomic layers with different chemical environmental characteristics, which aims to study the interactions between solute atoms and LPSO structures, and Figure 4 shows the calculated interaction energies.

The positive interaction energy indicates that solute atoms are repelled by *m* phases, and the negative interaction energy suggests that solute atoms are attracted by *m* phases. In the 4H phase, only 3 types of solute atoms (Fe, W, Bi) have positive interaction energies at the second and third layers, and 14 types of solute atoms (V, Cr, Mn, Co, Ni, Nb, Mo, Ag, Ba, Ta, Pt, Au, Ru, and Re) have positive interaction energies at the third layer, except for negative interaction energies at the second layer, while the remaining 27 types of solute atoms (Li, Be, B, Na, Al, Si, P, S, K, Ca, Sc, Ti, Cu, Zn, Ga, Ge, Se, Sr, Y, Zr, Cd, Sn, Sb, Te, Cs, Hf, and Pb) have negative interaction energies at the second and third layers, indicating that these solute atoms (Li, Be, B, Na, Al, Si, P, S, K, Ca, Sc, Ti, V, Cr, Mn, Co, Ni, Cu, Zn, Ga, Ge, Se, Sr, Y, Zr, Nb, Mo, Ag, Cd, Sn, Sb, Te, Cs, Ba, Hf, Ta, Pt, Au, Pb, Ru, and Re) are attracted by 4H phase. In the 6H phase, only a solute atom (Bi) has positive interaction energies at the first, second, third, and sixth layers, while the remaining 43 types of solute atoms have negative interaction energies at the first, second, third, or sixth layer, suggesting that all solute atoms except for the Bi atom are attracted by the 6H phase; noticeably, the interaction energies of the Zn and Y solute atoms show that they are attracted to the stacking fault planes of the 6H phase, which agrees well with the experimental observations by atomic-resolution Z-contrast STEM [20]. In the 8H phase, only 12 types of solute atoms (Li, Be, Na, Co, Ni, Cu, Zn, Nb, Ag, Pt, Au, and Bi) have positive interaction energies at the second, third, and fourth layers, while the remaining 32 types of solute atoms have negative interaction energies at the second, third, or fourth layers, implying that the 32 types of solute atoms are attracted by the 8H phase. In the 9R phase, only 6 types of solute atoms (Cr, Nb, Mo, W, Bi, and Re) have positive interaction energies at the second and third layers, while the remaining 38 types of solute atoms have negative interaction energies at the second or third layers, meaning that the 38 types of solute atoms are attracted by the 9R phase. In the 12H phase, only 17 types of solute atoms (Li, Be, Na, V, Cr, Fe, Co, Ni, Cu, Zn, Nb, Mo, Ag, W, Pt, Au, and Bi) have positive interaction energies at the second, third, fourth, and fifth layers, while the remaining 27 types of solute atoms have negative interaction energies at the second, third, fourth, or fifth layers, meaning that the 27 types of solute atoms are attracted by 12H phase. In the 15R phase, only 6 types of solute atoms (V, Cr, Nb, Mo, W, Bi) have positive interaction energies at the second, third, and fourth layers, while the remaining 38 types of solute atoms have negative interaction energies at the second, third, or fourth layers, signifying that the 38 types of solute atoms are attracted by the 15R phase; noticeably, the interaction energies of Co and Y solute atoms demonstrate that they are attracted to the stacking fault planes of the 15R phase, which agrees well with the experimental results [26]. In the 16H phase, only a solute atom (Bi) has positive interaction energies at the second, third, fourth, fifth, and sixth layers, while the remaining 43 types of solute atoms have negative interaction energies at the second, third, fourth, fifth, or sixth layers, suggesting that the 43 types of solute atoms are attracted by the 16H phase.

**Figure 5:**
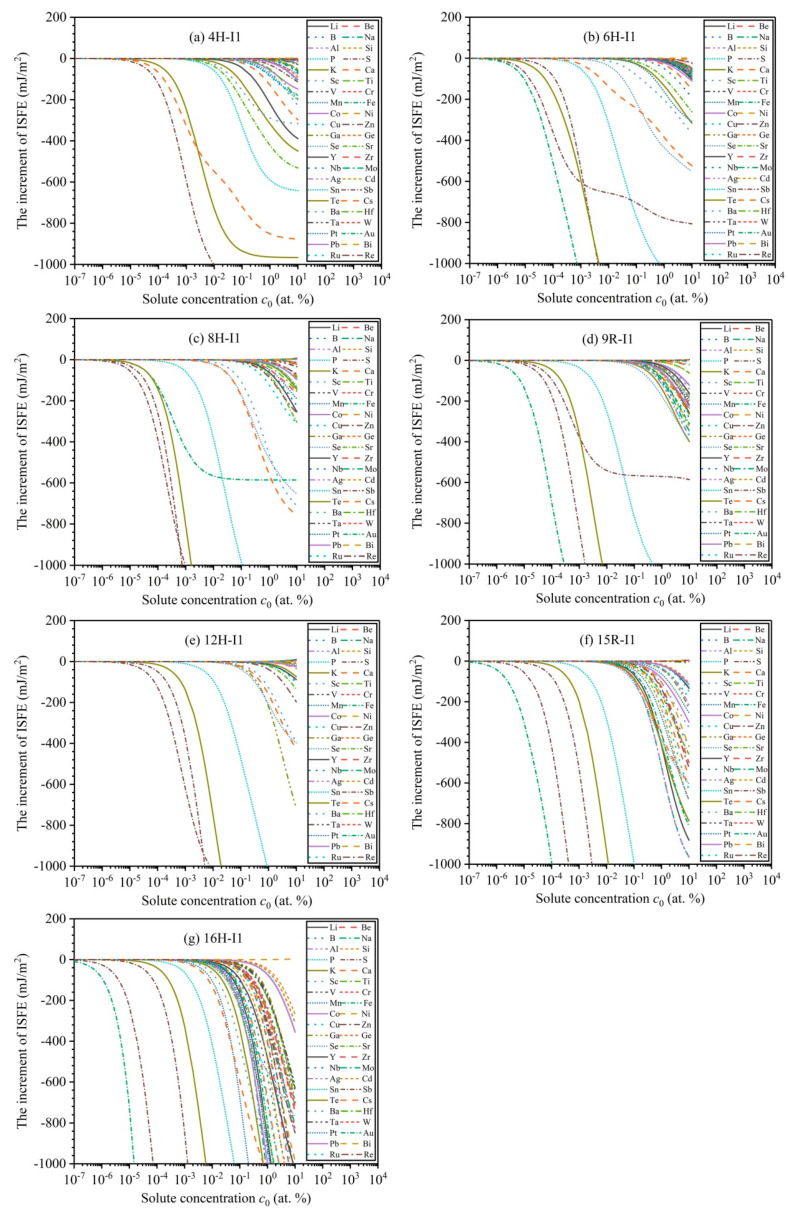
Variation curves of the increments of ISFEs as a function of solute concentrations $c_{0}$ at T=300 K: (**a**–**g**) denote 4H, 6H, 8H, 9R, 12H, 15R, and 16H phases, respectively.

In general, the stability of LPSO structures mainly depends on the magnitude of ISFEs. The low ISFEs improve the stabilities of LPSO structures, whereas the high ISFEs suppress the stabilities. Solute atoms play an important role in affecting the magnitude of ISFEs and then effectively promote their structural stabilizations, thus improving the mechanical properties of Mg alloys [42,43,44,45]. On the basis of the FDD model, Figure 5 demonstrates the dependencies of solute concentrations $c_{0}$ as a function of the increments of ISFEs at *T* = 300 K, and various alloying elements have different impacts on the increments of ISFEs of *m* phases. In the 4H phase, the three types of solute atoms (Sb, Te, Cs) significantly decrease the increments of ISFEs at $c_{0}<0.1\%$ compared with the other alloying elements, thus indicating that the three types of alloying elements are helpful to promote structural stabilizations of the 4H phase. In 6H, 8H, 9R, and 15R phases, the five types of solute atoms (P, S, Fe, Sb, and Te) remarkably decrease the increments of ISFEs at $c_{0}<0.1\%$ compared to the other alloying elements, thus suggesting that the five types of alloying elements are beneficial to promoting structural stabilizations of 6H, 8H, 9R, and 15R phases. In the 12H phase, the four types of solute atoms (P, S, Sb, and Te) markedly decrease the increments of ISFEs at $c_{0}<0.1\%$ compared with the other alloying elements, thus signifying that the four types of alloying elements are beneficial to promoting structural stabilizations of the 12H phase. In the 16H phase, the eight types of solute atoms (P, S, Mn, Fe, Sb, Te, Cs, and Ba) significantly reduce the increments of ISFEs at $c_{0}<0.1\%$ compared to the other alloying elements, meaning that the eight types of alloying elements contribute to promoting structural stabilizations of the 16H phase. Therefore, according to the above results, these alloying elements with significant promoting effects can become potential candidates for improving the mechanical properties of Mg alloys.

**Figure 6:**
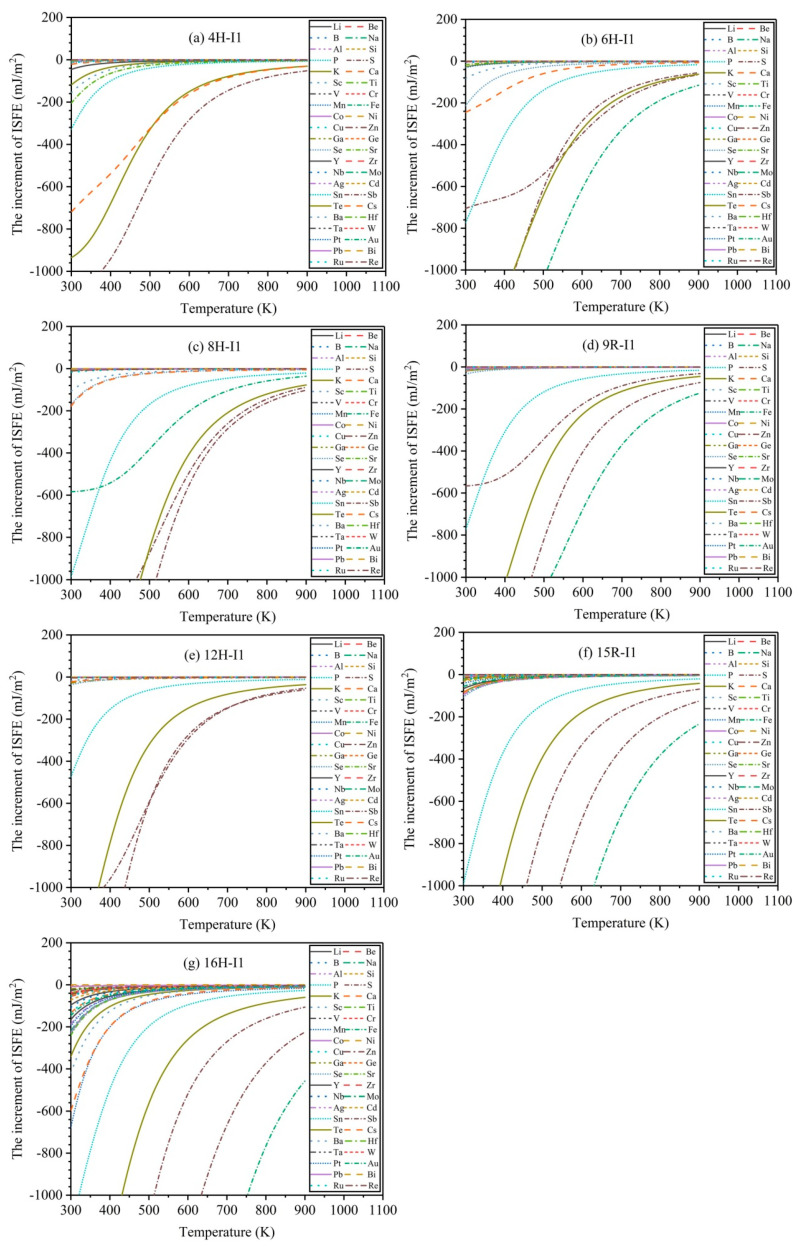
Variation curves of the increments of ISFEs as a function of finite temperature *T* at $c_{0}=0.1\%$: (**a**–**g**) denote 4H, 6H, 8H, 9R, 12H, 15R, and 16H phases, respectively.

To further explore the influences of finite temperature (T≤900 K) on the increment of ISFEs, Figure 6 exhibits the variation curves of the increments of ISFEs as a function of the finite temperature *T* at $c_{0}=0.1\%$ for 44 types of solute atoms. With the increase in temperature, the increments of ISFEs increase, indicating that high temperature inhibits the stabilities of *m* phases, because at high temperatures, smaller numbers of solute atoms are concentrated in the *m* phases according to the FDD model, and they give smaller contributions in the decrease in the ISFEs. In the 4H phase, the three types of solute atoms (Sb, Te, Cs) significantly decrease the increments of ISFEs with the increase in temperature (T≥700 K), and Sb atom has the strongest effect on decreasing the increments of ISFEs of 4H phase compared with Te and Cs atoms. In 6H, 8H, 9R, 15R, and 16H phases, the four types of solute atoms (S, Fe, Sb, and Te) dramatically reduce the increments of ISFEs with the increase in temperature (T≥700 K), and Fe atom has the strongest effect on decreasing the increments of ISFEs of the 6H, 9R, 15R, and 16H phases with regard to S, Sb, and Te atoms, and the Sb atom has the strongest effect on lowering the increments of ISFEs of the 8H phase compared with S, Fe, and Te atoms. In the 12H phase, the three types of solute atoms (S, Sb, and Te) markedly decrease the increments of ISFEs with the increase in temperature (T≥700 K), where S and Sb atoms have the strongest effects on lowering the increments of ISFEs of 12H phase with respect to the Te atom. Therefore, according to the above results, these potential alloying elements are helpful in promoting the stabilities of LPSO structures at high temperatures, improving mechanical properties of Mg alloys at high temperatures.

**Figure 7:**
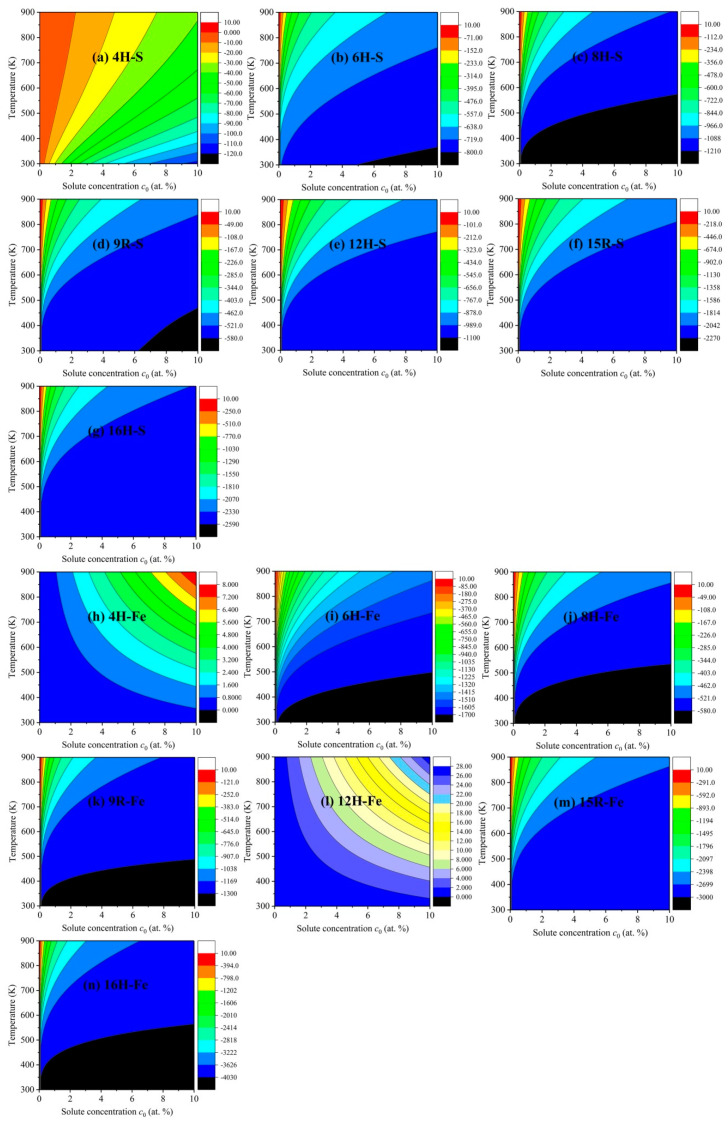
Two-dimensional diagrams of the increments of ISFEs versus solute concentration and temperature in *m* phases: (**a**–**g**) denote 4H, 6H, 8H, 9R, 12H, 15R, and 16H phases for the S atom, (**h**–**n**) indicate 4H, 6H, 8H, 9R, 12H, 15R, and 16H phases for the Fe atom, respectively.

To shed new light on the common influences of solute concentration and temperature on the increments of ISFEs, Figure 7 depicts the 2D diagrams of S and Fe atoms with strong effects, where Figure 7a–g show the common influences of the S solute atom, and Figure 7h–n exhibit the common influences of the Fe solute atom in *m* phases. For the S solute atom, the increments of ISFEs are decreased in the case of a certain solute concentration and temperature in the following order: 16H > 15R > 8H > 12H > 6H > 9R > 4H, suggesting that the S atom is the most likely to improve the stability of the 16H structure with respect to the other *m* phases. For the Fe solute atom, the increments of ISFEs are reduced at the same solute concentration and temperature in the following order: 16H > 15R > 6H > 9R > 8H, indicating that the Fe atom is the most likely to promote the stability of the 16H structure with respect to 6H, 8H, 9R, and 15R phases. Unfortunately, the Fe atom increases the increments of ISFEs in 4H and 12H phases at any solute concentration and temperature, thus meaning that doping the Fe atom tends to suppress structural stabilization of 4H and 12H phases, which can offer valuable references to further study and develop high-performance Mg alloys with LPSO structures.

## 5. Conclusions

In this work, we apply first-principle high-performance calculations to investigate the stabilization mechanisms of multi-type LPSO structures in Mg alloys and the effects of 44 types of elements from the periodic table of elements on the stabilities of LPSO structures. The formation enthalpies of solute atoms, as well as the interaction energies and increments of ISFEs are calculated. The relevant conclusions are summarized as follows:

(1) The LPSO structures for 9R, 12H, 15R, and 16H are essentially easy to form and exist stably in Mg metal, and the 15R phase is the easiest to form and stabilize among these LPSO structures, but the others for 4H, 6H, and 8H are comparatively difficult to form and exist stably owing to their relatively high ISFEs.
(2) The calculated results of interaction energies indicate that 44 types of solute atoms have different segregation characteristics in *m* phases, including attractions and repulsions by the SFs of *m* phases.
(3) These alloying elements, such as elements (Sb, Te, and Cs) for 4H, elements (P, S, Fe, Sb, and Te) for 6H, 8H, 9R, and 15R, elements (P, S, Sb, and Te) for 12H, and elements (P, S, Mn, Fe, Sb, Te, Cs, and Ba) for 16H, can markedly promote structural stabilizations of *m* phases and become potential candidates in improving mechanical properties of Mg alloys.
(4) A high temperature inhibits the stabilities of LPSO structures. These alloying elements, such as elements (Sb, Te, and Cs) for 4H, elements (S, Fe, Sb, and Te) for 6H, 8H, 9R, 15R, and 16H, and elements (S, Sb, and Te) for 12H, can effectively promote structural stabilizations of *m* phases at high temperature, improving mechanical properties of Mg alloys at high temperatures.
(5) Two-dimensional diagrams reveal that S and Fe atoms are the most likely to promote the stabilities of the 16H structure with respect to the other *m* phases, but the Fe atom tends to suppress structural stabilizations of the 4H and 12H phases.

## Data Availability

The data presented in this study are available from the corresponding author upon request.
